# Evaluating the efficacy of local anesthetic techniques during scalp microneedling

**DOI:** 10.1111/jocd.16480

**Published:** 2024-09-08

**Authors:** Szymon Leonik, Michał Smoczok, Beata Bergler‐Czop

**Affiliations:** ^1^ Department of Dermatology Medical University of Silesia Katowice Poland; ^2^ Department of Plastic, Reconstructive and Aesthetic Surgery Antoni Jurasz University Hospital Bydgoszcz Poland

**Keywords:** androgenetic alopecia, anesthesia, creams, microneedling, topical treatment

## Abstract

**Introduction:**

An increasing interest in minimally invasive procedures hassled to a demand for an effective local anesthetic algorithm. The scalp presents a challenge in achieving effective anesthesia due to the presence of hair shafts. This study aims to evaluate the efficacy of different methods during a microneedling procedure, including 25 mg lidocaine and 25 mg prilocaine cream, skin spray with 10% lidocaine, and cold gel compresses.

**Materials and Methods:**

Sixty‐two men aged between 20 and 50 years underwent three microneedling treatments, each using a different method of anesthesia. The treatment area was divided into two equal parts, with one part exposed to a specific anesthetic method. Patients were asked to rate their pain on a 0–10 verbal analog scale. An attempt was made to objectify the results using algometry.

**Results:**

A negative correlation was observed between the algometry results and the VAS score after the application of the cream and cold compresses.

**Discussion:**

When choosing monotherapy, it is recommended to use cold gel compresses for scalp microneedling after considering the advantages and disadvantages of different methods.

## 1 INTRODUCTION

For a patient about to undergo the procedure, it is important to consider not only the final result but also the comfort of the procedure itself. The increasing popularity and use of aesthetic medicine methods have led to a demand for an efficient and rapid method of anesthesia for the procedure. An example of a minimally invasive method is microneedling (MN). MN involves puncturing the skin's surface to create microchannels. This stimulates a regeneration cascade through the release of platelet‐derived growth factor, transforming growth factor alpha and beta, connective tissue activating protein, connective tissue growth factor and fibroblast growth factor. MN is a technique used to treat various skin problems, such as wrinkles, scars, and stretch marks.^1^, ^2^, ^3^, ^4^, ^5^, ^6^ Recent research has focused on the use of MN in treating androgenetic alopecia (AGA). Reports suggest that MN stimulates the Wnt/β‐catenin pathway, which is crucial for maintaining normal hair condition.^7^, ^8^ MN is popular due to its simplicity, affordability, and safety. The procedure is carried out until alocal redness and spot bleeding occur, and it may cause subjective severe pain. It is important to use an effective method of anesthesia both from the patient's and physician's point of view. An inadequate pain relief can cause unnecessary stress, increase treatment time, limit the range of treatments, and discourage patients from repeating them. The ideal topical pain control method should be non‐toxic, painless when applied, effective, fast‐acting, and carry a low risk of allergy.^9^ This article discusses non‐injectable pain control methods, such as 25 mg lidocaine and 25 mg prilocaine cream, skin spray with 10% lidocaine, and cold gel compresses.

## 2 MATERIALS AND METHODS

The study involved a group of 62 men between the age of 20 and 50 with features of androgenetic alopecia who underwent three MN treatments. A different type of anesthetic was used for each session. The treatment area was divided into two equal parts, one of which received local anesthetic. Subjective pain ratings for the area with and without anesthesia were assessed by the patients using the verbal analog scale (0–10) (VAS). Subjective pain was attempted to be objectified using an algometer. The scalp was divided into six sections and a blunt needle device was applied to each section at the last visit, exposing the skin to increasing pressure. The patient was asked to indicate the moment of pain intensity at 5 on the VAS scale. The arithmetic mean was calculated from the values obtained, expressed in grams. A cream containing 25 mg lidocaine and 25 mg prilocaine was applied to the scalp under occlusion 30 min before the procedure. The skin was then thoroughly cleansed of the cream. Three sprays were used for the 10% lidocaine aerosol. The dose used was equivalent to 13.8 mg of lidocaine. The preparation was left on the skin for 1 min, then the skin was cleansed. The pre‐prepared gel compress, wrapped in disposable foil at about −13°C, was applied to the cleansed skin for 90 s until a temperature of about 10°C was reached. In all cases, the same person applied the compress to minimize differences in pressure. The ambient temperature during the treatment was about 23°C. When the surface temperature of the treated skin returned, the compress was reapplied until the treatment was complete. Control measurements were taken with a thermal imaging camera every 10 s from a distance of approximately 50 cm. The Bioethics Committee has granted approval for the conduct of the study, designated as PCN/CBN/0052/KB1/10/22, dated March 8, 2022.

## 3 RESULTS

**FIGURE 1:**
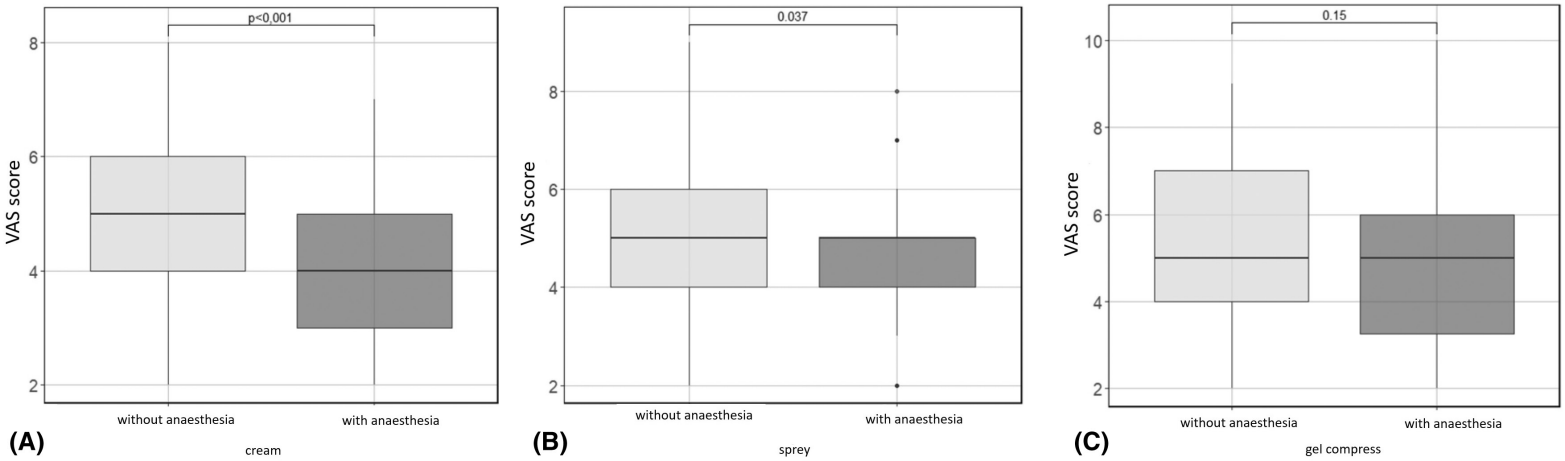
Summary of VAS scores before and after application of: (A) cream, (B) spray, (C) cold gel compresses. Wilcoxon test.

Table 1 summarizes the results. The mean VAS scale without cream anesthesia was significantly higher than the scale degrees after its application (*p* < 0.001). Similarly, the mean VAS scale without spray anesthesia was significantly higher than the scale steps after its application (*p* < 0.037). The use of gel packs did not significantly affect the VAS scale before and after anesthesia (Figure 1).

**TABLE 1:** VAS value after different types of anesthesia. Determined by the Wilcoxon test.

Visit	VAS Scale	*n*	Min	Max	Median	*q*1	*q*3	IQR	Mean	SD	*p*
1	Without anesthesia	62	2	8	5	4	6	2	5.23	1.40	<0.001
	After applying the cream	62	2	7	4	3	5	2	4.05	1.23	
2	Without anesthesia	62	2	9	5	4	6	2	5.39	1.60	0.037
	After using the spray	62	2	8	5	4	5	1	4.81	1.32	
3	Without anesthesia	62	2	9	5	4	7	3	5.26	1.72	0.147
	After applying gel compress	62	2	10	5	3.25	6	2.75	4.82	1.71	

Abbreviation: IQR, interquartile range; VAS, Verbal Analog Scale.

**FIGURE 2:**
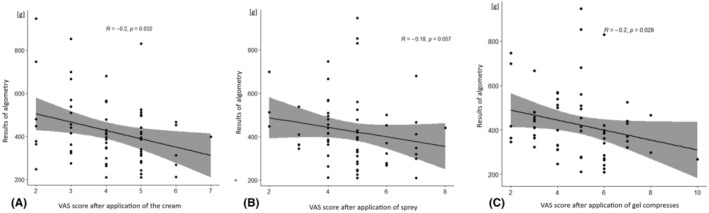
Correlation of mean algometry score with VAS after application of: (A) cream, (B) spray, (C) cold gel packs. Kendall's correlation.

The study found a negative correlation between algometry score and VRS when cream was used as the method of anesthesia. However, no correlation was observed between algometry score and VRS when spray was used. Additionally, a negative correlation was observed between algometry score and VRS when gel compresses were used as the method of anesthesia (Figure 2).

## 4 DISCUSSION

**FIGURE 3:**
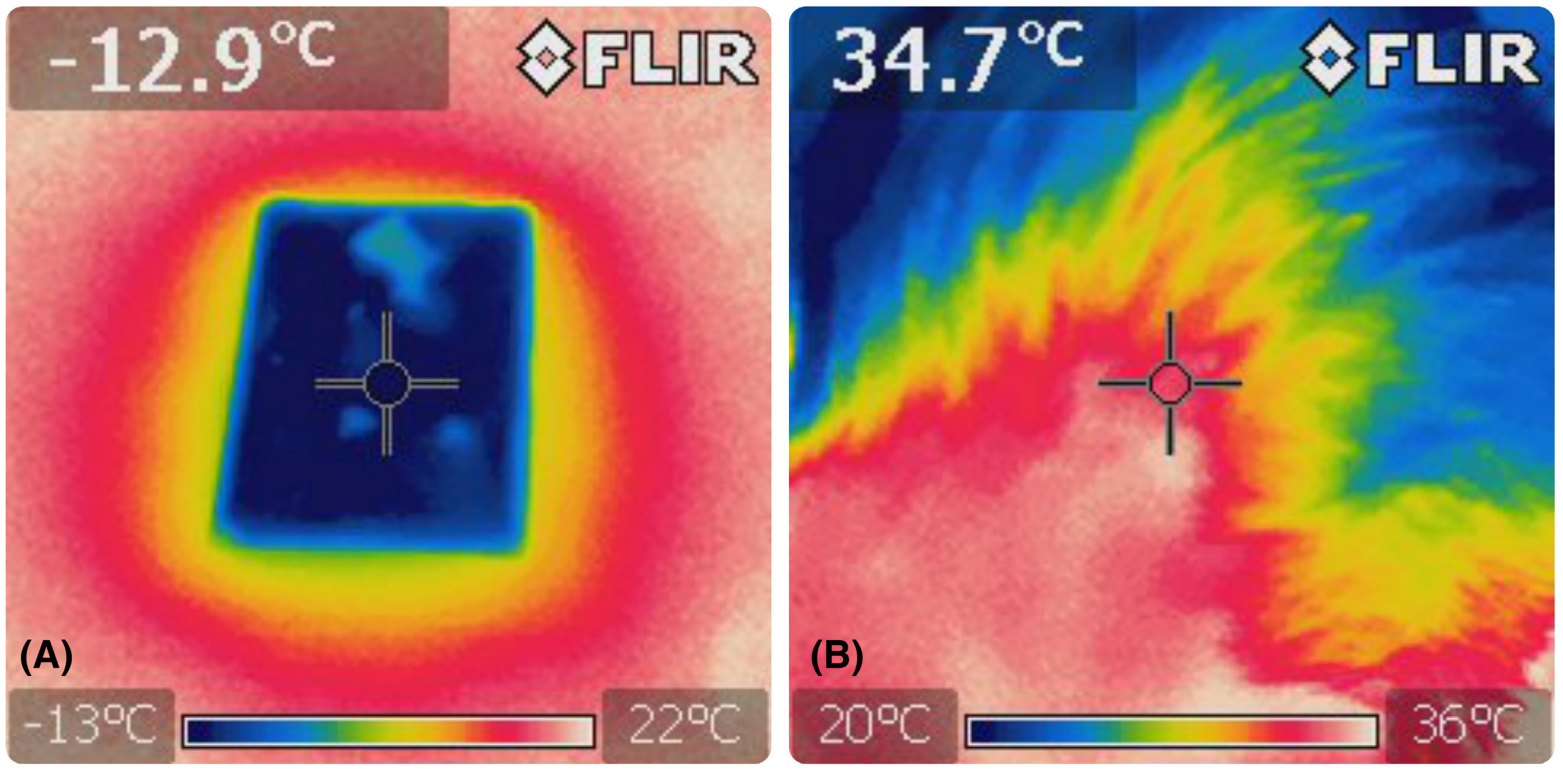
(A) Initial temperature of the cold gel compress. (B) Initial temperature of the treatment area.

**FIGURE 4:**
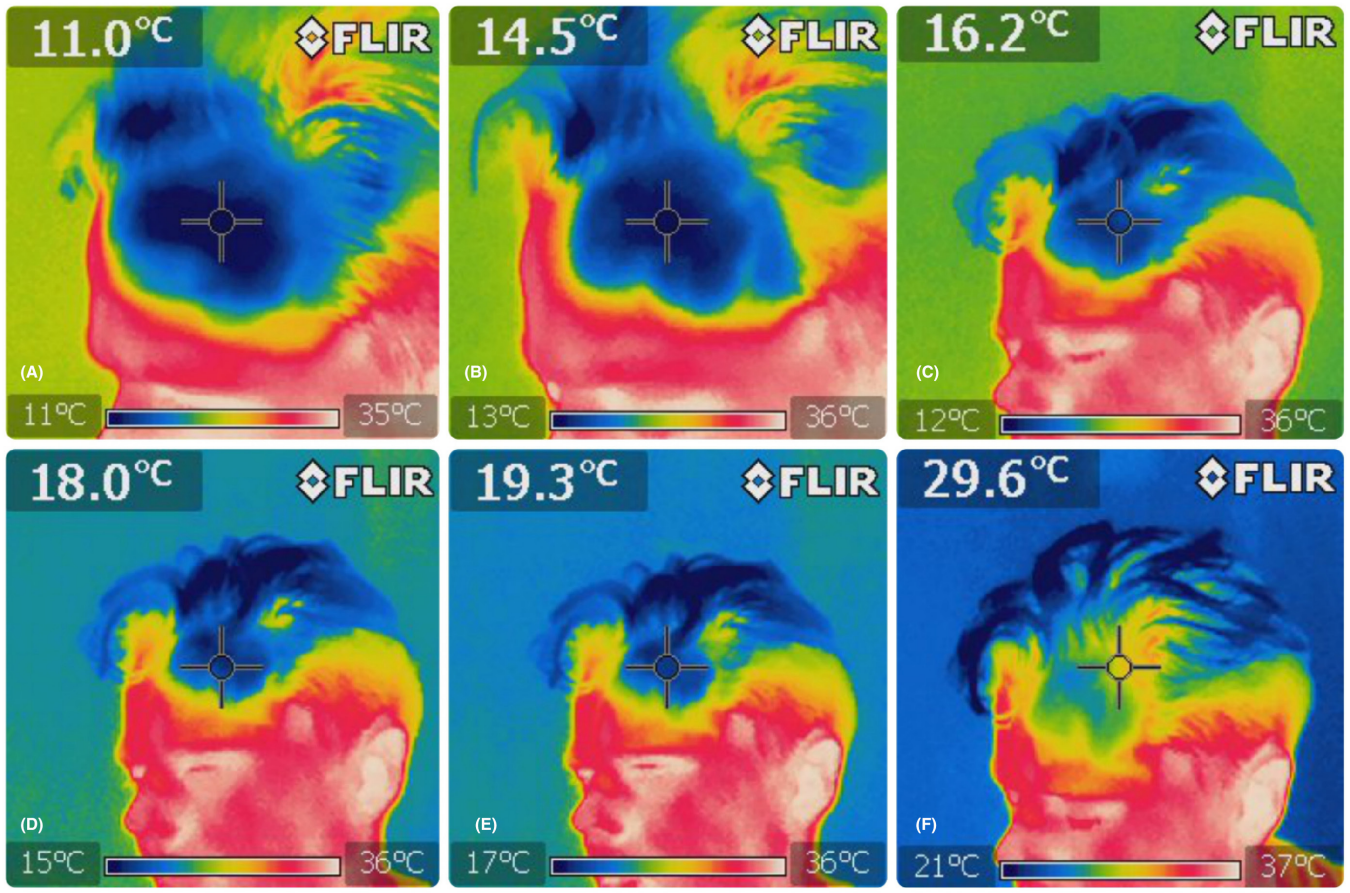
Surface temperature measurements of the treated scalp. (A) Measurement immediately after application of the cold compress, (B–E) measurements every 10 s after application of the compress. The time of the assumed range from 10 to 20°C was about 50 s. (F) A temperature of approximately 30°C was obtained approximately 2 min after the application of the compress.

The available anesthetic creams differ in their ingredients, leading to varying pharmacological characteristics, profiles, and durations of action.^10^ For this study, we used the most common formulation. However, it is important to note that all formulations have one disadvantage in common: they are difficult to wash off the scalp before treatment. This method is not recommended for people with longer hair due to the fact that intense rubbing can induce hair loss, be time‐consuming, and can be unpleasant for the patient.^11^ Additionally, the length of time the cream must remain on the skin makes it virtually impossible to apply a repeat dose of anesthesia if the patient reports severe pain. Therefore, we recommend that this method should not be the default for scalp MN. The aerosol is known for its fast action and being easy to use. However, it contains 96% ethanol, which can irritate the skin.^12^ This is very important if further doses need to be administered during MN. Cold gel packs meet many of the criteria for ideal local pain control: they are inexpensive, fast acting, non‐toxic and do not carry the risk of sensitization. The vasoconstrictive effect of cold should also be considered. This phenomenon, which is desirable in other treatments for MN, can make it difficult to determine when the treatment has been finished in a particular area. The vasoconstrictive effect is evident from around 15°C. The aim to reducing the temperature was to decrease nerve conduction and thus increase the pain threshold. When the skin temperature drops to 10°C, the nerve conduction velocity decreases by about 33%.^9^, ^13^, ^14^, ^15^ Based on the information provided, it is recommended to maintain a temperature range of 10–20°C during the MN procedure. This range allows for initial anesthesia, efficient procedure performance, and clear observation of the end point. It is important to note that this method may require multiple applications during a single procedure. Additionally, the use of low temperatures may cause discomfort.^16^ During the study, 4 out of 62 patients (6.45%) reported experiencing subjective pain levels exceeding 4/10 on the VRS scale (0–10 scale, where ≥4 indicates moderate pain) at a superficial temperature of approximately 14°C. This information should be considered when planning the procedure and discussing it with the patient (Figures 3 and 4).

Based on the data presented, it is believed that cold gel compresses are the most justified choice for monotherapy. Despite its disadvantages, this method appears to be the most effective for MN on hairy skin due to its speed, lack of risk of allergy and irritation. A promising method, not studied in this research but recommended in publications, is the use of vibration as a non‐injectable method.^16^, ^17^, ^18^, ^19^ It should be considered in future analyses.

## CONFLICT OF INTEREST STATEMENT

The authors declare no conflicts of interest.

## ETHICS STATEMENT

The research was acknowledged by the bioethics committee, as indicated by the corresponding number provided in the Materials and Methods section.

## Data Availability

The data that support the findings of this study are available from the corresponding author upon reasonable request.
